# Partial Fanconi Syndrome Induced by Ifosfamide

**DOI:** 10.7759/cureus.3947

**Published:** 2019-01-23

**Authors:** Sulagna Das, Damian N Valencia, Adam Fershko

**Affiliations:** 1 Internal Medicine, Kettering Medical Center, Dayton, USA

**Keywords:** fanconi syndrome, ifosfamide, renal tubular acidosis, rta

## Abstract

Several commonly used chemotherapeutic agents, antibiotics, antivirals, and antiepileptic medications can cause partial or full Fanconi syndrome, disorders which can generally be described as transport defects in the proximal renal tubule, associated with non-anion gap metabolic acidosis. Fanconi syndrome is underreported and therefore often missed in the clinical setting. Herein, we present a case report that details the course of a 64-year-old female with a history of stage IV undifferentiated pleomorphic sarcoma who after her sixth chemotherapeutic cycle (adriamycin, ifosfamide, and mesna) developed severe hypokalemia, hypophosphatemia, and proteinuria without glycosuria, eventually diagnosed with partial Fanconi syndrome. The aim of this report is to highlight the importance of routine serum and urine monitoring in patients undergoing therapy with potentially nephrotoxic agents to avoid potentially fatal renal nephrotoxicity, including partial and full Fanconi syndrome.

## Introduction

Several commonly used chemotherapeutic agents, antibiotics, antivirals, and anti-epileptic medications can cause partial or full Fanconi syndrome. Fanconi syndrome is a metabolic disorder, which was first described by Lignac in 1924 and later defined by Fanconi in 1936 [1-3]. It is characterized by dysfunction of the proximal renal tubules, leading to impaired proximal tubular reabsorption of amino acids, glucose, phosphate, urate, and bicarbonate. This results in profound bone loss resistant to vitamin-D therapy, commonly rickets or osteomalacia [4]. Ifosfamide is a widely used chemotherapeutic agent with known renal toxicity, specifically altering filtration at the proximal tubule [5-8]. Toxicity arises from tubular cell organic cation transporter-mediated uptake and metabolism of ifosfamide into chloroacetaldehyde [5,9]. Ifosfamide-induced nephrotoxicity, proximal tubulopathy, and glomerulopathy were first described in 1972 [10]. Although ifosfamide toxicity is often associated with hypokalemia and diabetes insipidus, more worrisome complications include acute renal failure and severe bone demineralization from urinary phosphate loss, either through partial or full Fanconi syndrome. Herein, we discuss a case of metastatic undifferentiated pleomorphic sarcoma treated with combination chemotherapy (adriamycin, ifosfamide, mesna) with the subsequent development of partial Fanconi syndrome, illustrating both diagnostic features and therapeutic options.

## Case presentation

A 64-year-old, African American female, with a history of metastatic poorly differentiated pleomorphic sarcoma of the right thigh and hypertension, presented to the emergency department with concerns over increased fatigue and generalized weakness which began 10 days ago, after a scheduled chemotherapy session with adriamycin, ifosfamide, and mensa. The patient also reported difficulty with ambulation and inability to accomplish full range of motion in the upper extremities against gravity. Associated symptoms included polyuria and polydipsia. On review of systems, the patient denied chest pain, shortness of breath, nausea, vomiting, diarrhea, abdominal pain, fevers, chills, night sweats, reduction in appetite, and weight loss. On further review of oncologic history, it appeared that the patient received neoadjuvant radiation therapy to the right thigh, followed by surgical resection. Unfortunately, the patient was later diagnosed with metastatic lesions in the lungs and was subsequently treated with six cycles of palliative chemotherapy (adriamycin, ifosfamide, and mesna). The total cumulative dose of ifosfamide was 45 g/m^2^. The patient also experienced severe neutropenia requiring a 25% dose reduction of adriamycin and ifosfamide during the second cycle of therapy. After the third cycle, imaging studies revealed disease stabilization. Prior to presentation, weekly laboratory studies revealed normal sodium, potassium, bicarbonate, creatinine, and phosphorus levels. On initial evaluation, the patient was hemodynamically stable; electrocardiogram (EKG) showed normal sinus rhythm and chest X-ray showed no pathologic cardiopulmonary process. Laboratory studies revealed a non-anion gap metabolic acidosis and acute kidney injury; markedly abnormal laboratory values include 152 mmol/L of sodium, 1.3 mmol/L of potassium, 16 mmol/L of bicarbonate, 1.1 mg/dL of phosphorus, 1.8 mg/dL of magnesium, and 3.3 mg/dL of creatinine. The blood glucose level was 123 mg/dL. The complete serologic data are listed in Table 1. 

**Table 1:** Serum Biochemical Parameters

Serum Biochemical Parameters	Patient's Value	Age-adjusted Reference Range
Hemoglobin (g/dL)	6.1	12.1-15.8
Hematocrit (%)	18.4	35.8-46.5
Fibrinogen (mg/dL)	568	169-404
D-Dimer (ng/mL)	252	0-241
pCO2 (mmHg)	22	34-45
Sodium (mEq/L)	151	135-145
Potassium (mEq, L)	1.5	3.5-5.0
Chloride (mEq/L)	121	96-106
Bicarbonate (mEq/L)	16	22-28
Anion Gap (mmol/L)	8	7-16
Glucose (mg/dL)	101	74- 106
Blood Urea Nitrogen (mg/dL)	32	15-40
Creatinine (mg/dL)	3.2	0.2-0.7
Calcium (mg/dL)	7.9	8.0-10.5
Phosphorus (mg/dL)	1.1	3.8-6.2
Albumin	2.7	3.4-5.0
Lactate dehydrogenase	181	84-246
Magnesium (mg/dL)	1.7	1.5-2.3

Urinalysis demonstrated a specific gravity of 1.012, protein level of 100 mg/dL, pH 6.0, and 20-50 epithelial cells per hpf. The complete urinalysis data are presented in Table 2. 

**Table 2:** Urine Biochemical Parameters

Urine Biochemical Parameters	Patient's Value	Age-Adjusted Reference Range
pH	6.0	5.0-8.0
Specific gravity	1.012	1.001-1.035
Protein (mg/L)	100	Negative
Sodium (mmol/L)	48	20
Potassium (mmol/L)	33.5	25-125
Glucose (mg/dL)	negative	negative
Epithelial Cells (/hpf)	20-50	0-3
WBC (/hpf)	3-5	0-3
RBC (/hpf)	3-5	0-3

After aggressive electrolyte repletion and intravenous fluid rehydration with normal saline, the patient experienced gradual improvement in weakness. The patient was soon after discharged with daily oral potassium supplementation. Chemotherapy has since been discontinued and electrolyte levels have remained stable.

## Discussion

The mechanism by which ifosfamide causes renal tubular damage is not completely understood. It is postulated that the metabolites of the medication, mainly chloroacetaldehyde and acrolein, are responsible for ifosfamide-induced Fanconi syndrome. Chloroacetaldehyde is known to be capable of depleting intracellular glutathione in the renal tubule, predisposing it to cellular damage [11]. Mesna, a water-soluble antioxidant, is often given for urinary bladder protection as it binds to acrolein and other urotoxic metabolites, preventing cellular damage. Intracellular depletion often results from the conversion of dimesna into mesna after reabsorption in the proximal renal tubule, where ifosfamide metabolism also induces glutathione depletion in the renal cortical cells. In combination, mesna administration during ifosfamide therapy may potentiate proximal tubule injury [12]. Proximal tubular reabsorption of glucose, phosphate, and sulfate require secondary sodium-coupled active transporters, which use solute-specific carrier proteins and an electrochemical sodium gradient created by Na-K-ATPase [13]. If this process is impaired, alteration in membrane permeability can disrupt transport protein function, impair mitochondrial adenosine triphosphate (ATP) production, and can inhibit the Na-K-ATPase pump. These transport defects caused by chloroacetaldehyde are similar to those of maleic acid-induced Fanconi syndrome, which are both associated with the direct impairment of Na-K pump activity and a decline of ATP generation in the proximal convoluted tubule [14-15].

In the case reported, the patient developed partial Fanconi syndrome after receiving a total of 45 g/m^2^ of ifosfamide without any additional risk factors. Factors that may influence the development of Fanconi syndrome are young age (less than 10 years old), prior cisplatin therapy, and pre-existing renal impairments such as chronic kidney disease or history of nephrectomy. Not to be mistaken for Fanconi syndrome, proximal renal tubular acidosis (RTA), also known as Type 2 RTA, is characterized by a decreased rate of bicarbonate reabsorption in the proximal renal tubule in the absence of decreased transport of other solutes. By contrast, Fanconi syndrome involves a generalized dysfunction at the proximal tubules, causing mass loss of electrolytes and proteins. Although these two disease processes are not mutually exclusive, as Fanconi syndrome does include proximal tubular acidosis through the loss of bicarbonate and may initially present as a type 2 RTA, differentiation is important as eventual disease pathology does differ. Severe bone loss resistant to vitamin-D therapy can be life-threatening and should be expected with true Fanconi syndrome. Therefore, it is imperative that clinicians monitor for serum (sodium, bicarbonate, calcium, magnesium, and phosphorus) and urine (glucose and protein) studies both during and after therapy with ifosfamide.

The case reported details a patient with partial Fanconi syndrome, as the patient experienced severe hypokalemia, proteinuria, and hypophosphatemia with a non-anion gap metabolic acidosis without glycosuria or vitamin-D-resistant bone disease. We believe early detection and treatment prevented further renal deterioration and the development of full Fanconi syndrome. Although uncommon, Fanconi syndrome can be fatal and should be considered in any patient with any degree of renal impairment. Although there is no definitive treatment, supportive measures with oral or intravenous fluid hydration and electrolyte repletion are the mainstay [16-18]. Some non-human studies suggest that oral supplementation with taurine or L-Histidinol can be renal protective in the setting of ifosfamide therapy via preservation of glutathione synthetase peroxidase and anti-oxidase systems [19-20]. Many other antioxidants (N-acetylcysteine, resveratrol, melatonin, L-carnitine, and thymoquinone) may have similar effects although further research is necessary before considering them as therapeutic options. 

## Conclusions

While the mechanism of ifosfamide-induced Fanconi syndrome is still unclear, it is imperative that clinicians be aware of its potential complications and monitor serum electrolytes, urine electrolytes, and protein/amino acid levels throughout ifosfamide therapy for early identification and management of nephrotoxicity.
